# EphB4 forward signalling regulates lymphatic valve development

**DOI:** 10.1038/ncomms7625

**Published:** 2015-04-13

**Authors:** Gu Zhang, John Brady, Wei-Ching Liang, Yan Wu, Mark Henkemeyer, Minhong Yan

**Affiliations:** 1 Department of Molecular Oncology, Division of Research, Genentech Inc., 1 DNA Way, South San Francisco, CA 94080, USA; 2 Department of Antibody Engineering, Division of Research, Genentech Inc., 1 DNA Way, South San Francisco, California 94080, USA; 3 Department of Developmental Biology, University of Texas Southwestern Medical Center, Dallas, Texas 75390, USA

## Abstract

Bidirectional signalling is regarded as a notable hallmark of the Eph-ephrin signalling system: Eph-dependent forward signalling in Eph-expressing cells and ephrin-dependent reverse signalling in Ephrin-expressing cells. The notion of ephrin-dependent reverse signalling derives from genetic experiments utilizing mice carrying mutations in the intracellular region of ephrinBs. Here we show that EphB4-dependent forward signalling regulates lymphatic valve development, a process previously thought to be regulated by ephrinB2-dependent reverse signalling. We develop antibodies that selectively target EphB4 and ephrinB2. We find that mice bearing genetically altered cytoplasmic region of ephrinB2 have significantly altered EphB4-dependent forward signalling. Selective inhibition of EphB4 using a functional blocking antibody results in defective lymphatic valve development. Furthermore, a chemical genetic approach is used to unequivocally show that the kinase activity of EphB4 is essential for lymphatic valve development.

## Abstract (web summary)

The bidirectional Eph-ephrin signalling regulates a myriad of developmental programmes. Zhang *et al*. show that EphB4 forward signalling is crucial for lymphatic valve development, providing new insight into this important developmental process previously thought to be regulated by ephrinB2-dependent reverse signalling.

## Graphical abstract

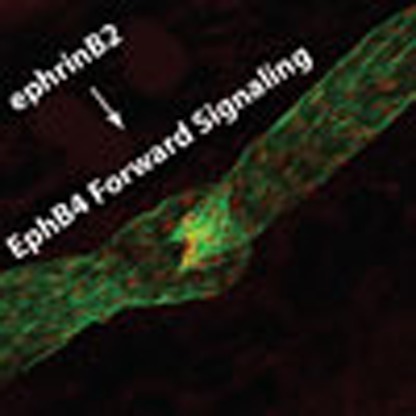

One important function of the lymphatic vasculature is to maintain tissue fluid homeostasis. Interstitial fluid is drained by the blind-ended lymphatic capillaries, transported by the collecting lymphatic vessels, and finally enters the venous circulation system via the thoracic duct^1^,^2^. A distinguishing hallmark for collecting lymphatic vessels is the presence of intraluminal lymphatic valves that are critical for preventing the lymph backflow. Genetic studies have revealed important regulators for lymphatic valve morphogenesis, including FOXC2, Connexin37, Connexin43, NFATc1, EphrinB2, integrin a9 and its ECM ligand Fibronectin-EIIIA, Semaphorin3A, Neuropilin-1, PlexinA1 and BMP9 (refs 3, 4, 5, 6, 7, 8, 9, 10, 11).

The Eph family of receptor tyrosine kinases and their membrane-anchored ephrin ligands possess versatile functions in regulating myriad developmental processes^12^. Eph-ephrin signalling involves multiple modes and mechanisms^13^. Besides the *trans* interaction between Eph receptors and ephrin ligands expressed on neighbouring cells, *cis* interactions can also occur between receptors and ligands expressed in the same cells. Endocytosis following receptor–ligand interaction also plays critical a role in determining the diverse outcomes of Eph-ephrin signalling. In addition, interplay with other signalling pathways is another important feature of Eph-ephrin signalling mechanism. The best example is the involvement of ephrinB2 in controlling vascular endothelial growth factor signalling^14^,^15^.

Bidirectional signalling is regarded as a notable hallmark of the Eph-ephrin signalling system: Eph-dependent forward signalling in Eph-expressing cells and ephrin-dependent reverse signalling in ephrin-expressing cells^16^. The notion of ephrin-dependent reverse signalling has been derived from genetic experiments utilizing mice carrying mutations in the intracellular region of ephrinBs^9^,^17^,^18^,^19^,^20^,^21^,^22^.

Here, we have gained important new insight into the role of EphB4-ephrinB2 signalling in lymphatic valve development, a process previously thought to be regulated by ephrinB2-dependent reverse signalling^9^,^23^. We have developed agonistic and antagonistic antibodies that selectively target EphB4 and ephrinB2, respectively. Using these antibodies in conjunction with mutant mice bearing genetically altered cytoplasmic region of ephrinB2, we find that ephrinB2-reverse signalling is dispensable. The mutant alleles of ephrinB2 are in fact either hypomorphic or hypermorphic with respect to activating EphB4-dependent forward signalling. Furthermore, a chemical genetic approach is used to unequivocally show that the kinase activity of EphB4 is essential for lymphatic valve development.

## Results

### Generating antibodies selectively targeting ephrinB2 and EphB4

**Figure 1:**
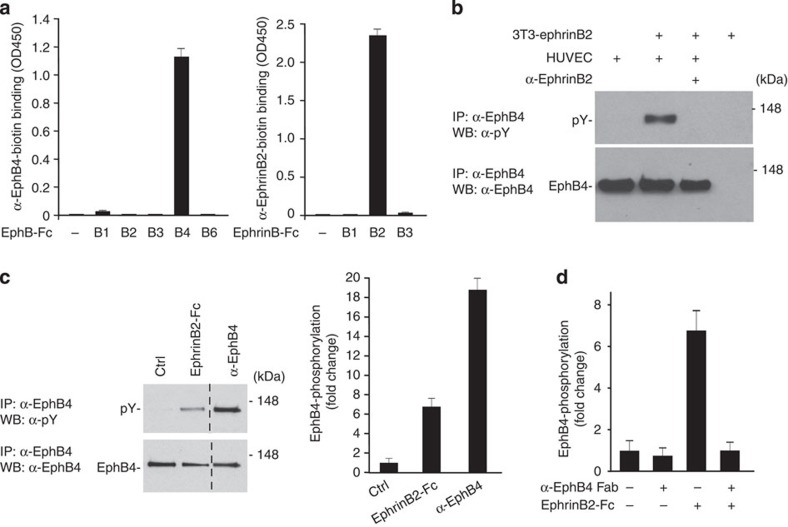
Characterization of anti-ephrinB2 and anti-EphB4 antibodies. (**a**) Biotinylated α-EphB4 selectively binds to EphB4-Fc, but not other EphB proteins (left); and biotinylated α-ephrinB2 selectively binds to ephrinB2-Fc but not to other ephrinB proteins (right). (**b**) Blocking activity of α-ephrinB2 measured by western blot (WB) analysis of EphB4 phosphorylation in HUVECs stimulated by overlaid ephrinB2-expressing 3T3 cells. (**c**) Agonistic activity of α-EphB4 measured by WB (left) and ELISA (right) of EphB4 phosphorylation in EphB4-expressing 3T3 cells treated with ephrinB2-Fc or α-EphB4. Dotted line indicates a cropped lane (full WB data in Supplementary Fig. 11). (**d**) Antagonistic activity of α-EphB4 Fab measured by ELISA of EphB4 phosphorylation in EphB4-expressing 3T3 cells. (**a**,**c**,**d**) Error bars, s.d. of technical triplicates. Ctrl, control; IP, immunoprecipitation.

Protein-null mutations of EphB4 or ephrinB2 in mice result in embryonic lethality due to vascular defect^14^,^24^,^25^. To facilitate postnatal functional assessment, we used phage display to generate high-affinity antibodies that selectively target EphB4 and ephrinB2 (Fig. 1a and Supplementary Fig. 1). An anti-ephrinB2 antibody was generated based on its ability to block ephrinB2 interaction with its receptor EphB4 (Supplementary Fig. 1c). It effectively inhibited EphB4 phosphorylation in human umbilical vein endothelial cells (HUVECs) overlaid with ephrinB2-expressing 3T3 cells (Fig. 1b). An anti-EphB4 antibody was also identified for its ability to block EphB4–ephrinB2 interaction (Supplementary Fig. 1d). Interestingly, anti-EphB4 acted agonistically to enhance EphB4 phosphorylation (Fig. 1c). The Fab fragment of anti-EphB4, however, failed to do so, indicating that the agonistic activity of anti-EphB4 depends on its bivalency. Anti-EphB4 Fab was able to block EphB4 phosphorylation induced by ephrinB2-Fc, consistent with its ability block the interaction between EphB4 and ephrinB2 (Fig. 1d and Supplementary Fig. 1d).

### Blockade of ephrinB2 causes lymphatic valve defects

**Figure 2:**
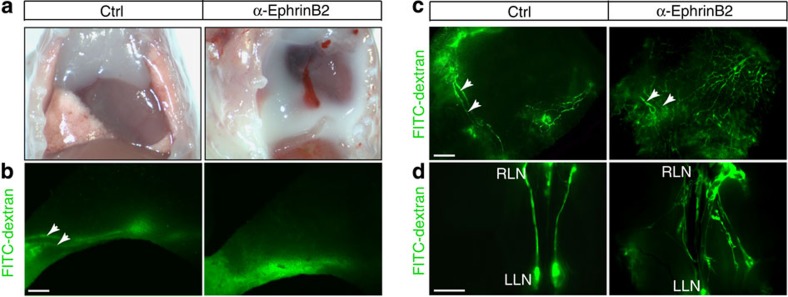
Anti-ephrinB2 causes dramatic lymphatic defects in neonatal mice. Treatment was started at P1 and mice were examined on P7. Scale bar, 1 mm. (**a**) Chylothorax in α-ephrinB2-treated mice. Exterior (**b**) and interior (**c**) views of the leg skins following injection of FITC-dextran into the hindlimb footpad. The main lymphatic collecting vessels are marked (arrowheads). Anti-ephrinB2 (α-ephrinB2)-treated animals exhibited abnormal outflow of FITC-dextran from collecting vessels to the pre-collector vessel branches. (**d**) Collecting vessels running between the lumbar (LLN) lymph nodes and renal lymph nodes (RLN). In control (Ctrl) mice, only a pair of collecting lymphatic vessel trunks was highlighted by FITC-dextran (left). In anti -ephrinB2-treated animals (right), FITC-dextran visibly diffused into the surrounding lymphatic network.

The antibodies we have generated target both human and mouse orthologues, allowing us to assess their *in vivo* activities in mouse models. Neonatal mice dosed with anti-ephrinB2 at postnatal day 1 (P1) usually (90%) died by P8. Examination of anti-EphrinB2-treated animals revealed apparent chylothorax, a condition where chyle from the thoracic duct effuses into the pleural space (Fig. 2a), indicating compromised lymphatic vasculature. Assessment of lymphatic function by examining the uptake and transport of large-molecule-weight fluorescent dye further confirmed the lymphatic defects (Fig. 2b–d).

**Figure 3:**
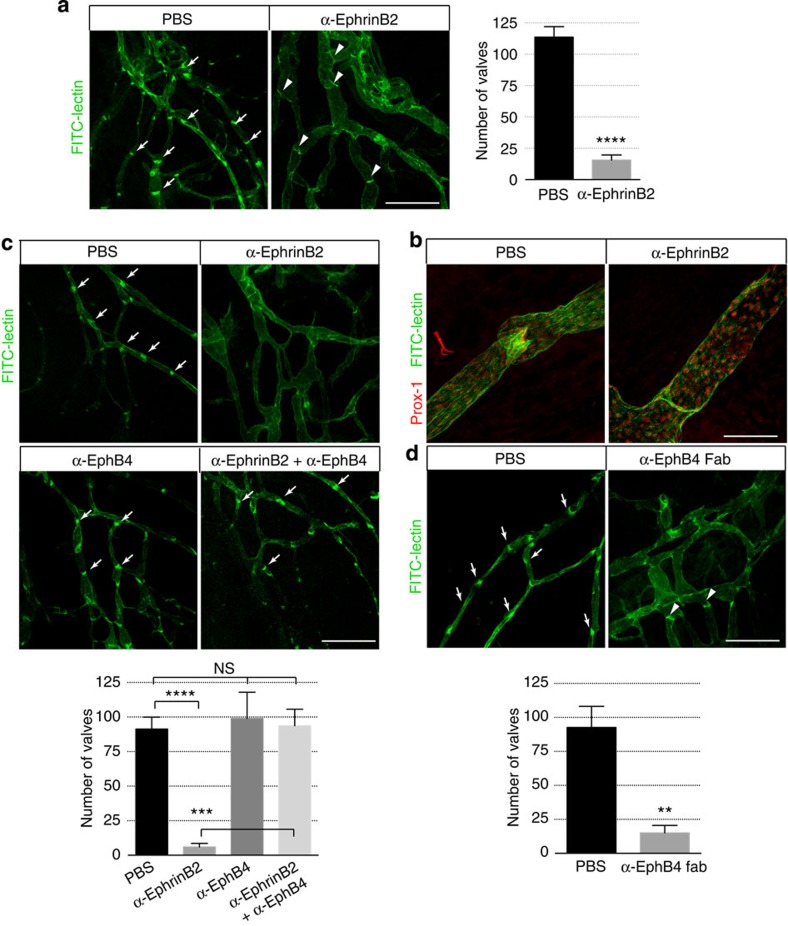
Effect of anti-ephrinB2 and anti-EphB4 antibodies on lymphatic valves. Visualization of lymphatic vessels and valves (arrows, not all valves are marked) by footpad injection of FITC-lectin following antibody administration from P1. (**a**) Defective lymphatic valves with ring-like appearance (arrow heads) in P5 leg skins of mice treated with function-blocking anti-ephrinB2 (α-ephrinB2) antibody. Right panel, quantification of lymphatic valves. (**b**) Higher magnification view of defective lymphatic valves in P5 leg skins of mice treated with function-blocking α-ephrinB2 antibody. (**c**) Agonistic anti-EphB4 (α-EphB4) reverses lymphatic valve defects caused by function-blocking α-ephrinB2. P6 leg skins of neonatal mice are shown. Lower panel, quantification of lymphatic valves. (**d**) Abnormal lymphatic valves with ring-like appearance (arrow heads) in P7 leg skins of mice treated with antagonistic anti-EphB4 Fab (α-EphB4 Fab). Lower panel, quantification of lymphatic valves. Scale bars: (**a**), (**c**) and (**d**), 500 μm; (**b**), 100 μm. ****P< 0.0001, ***P< 0.001, **P< 0.01 (two-tailed, unpaired Student's *t*-test), *n*=3 per treatment group (error bars, s.d.). NS, not significant.

Unidirectional lymphatic flow requires functional luminal valves^3^. At birth, mice have developed well-structured lymphatic valves. To evaluate the impact of ephrinB2 inhibition on lymphatic valves, we started anti-ephrinB2 treatment at P1 and examined the lymphatic collecting vessels between P2 and P7. FITC-lectin injected into the hindlimb footpad normally drains into the collecting lymphatic vessels in leg skins. FITC-lectin binds the surface of lymphatic endothelial cells and distinctively highlights the valves. At P5, lymphatic vessels in the leg skins of anti-ephrinB2-treated mice were apparently irregular and dilated (Fig. 3a). The characteristic V-shaped lymphatic valves were mostly absent. The remaining valves displayed a rudimentary morphology with a ring-shaped structure lacking the typical leaflets (Fig. 3a,b). The severity of lymphatic changes depended on the duration of ephrinB2 inhibition. At later time points, the lymphatic valves were almost completely lost (Fig. 3c). We also examined the mesenteric lymphatic vessels by immunostaining for Prox1, which readily revealed the lymphatic valves in control but not anti-ephrinB2-treated mice (Supplementary Fig. 2a). The lymphatic defects caused by anti-ephrinB2 are reminiscent of what have been described in mice that express a mutant ephrinB2 lacking the C-terminal PDZ interaction site (*ephrinB2*^ΔV/ΔV^). Similar to what has been observed in *ephrinB2*^ΔV/ΔV^ neonates^9^, anti-ephrinB2 antibody also caused changes in lymphatic remodelling. We found a significant increase of smooth muscle cell coverage in lymphatic capillaries (Supplementary Fig. 3). Interestingly, treatment of adult animals with anti-ephrinB2 for 2 weeks was well tolerated. There was no sign of chylothorax that observed in neonatal mice after anti-ephrinB2 treatment. In addition, the lymphatic valves were not affected (Supplementary Fig. 4), suggesting that ephrinB2 signalling is not required for lymphatic valve maintenance in adult mice.

### Agonistic anti-EphB4 negates the effect of ephrinB2 blockade

Although it has become evident that ephrinB2 is required for lymphatic valve development^9^,^23^, mechanistically, it remains unclear whether this function of ephrinB2 relies on its interaction with its receptor(s). During embryonic blood vascular development, EphB4 serves as the cognate receptor for ephrinB2 (refs 14, 25). To address whether EphB4 is also involved in regulating lymphatic development, we took advantage of the EphB4 antibody that selectively blocks the interaction between EphB4 and ephrinB2. Administration of anti-EphB4 in neonatal mice was well tolerated, in sharp contrast to the lethality caused by anti-ephrinB2. In addition, in anti-EphB4-treated animals, although the lymphatic vessels appeared to be slightly dilated, the lymphatic valves were abundantly present (Fig. 3c). Furthermore, co-administration of anti-EphB4 completely rescued the lethality caused by anti-ephrinB2, and reversed the lymphatic valve defects resulting from blocking ephrinB2 alone (Fig. 3c and Supplementary Fig. 2a). As anti-EphB4 is agonistic in stimulating EphB4 phosphorylation, these findings suggested that activation of EphB4 *in vivo* is sufficient to overcome the blockade of ephrinB2 by anti-ephrinB2. Consistent with its antagonistic activity against EphB4 signalling *in vitro* (Fig. 1d), the Fab fragment of anti-EphB4 caused marked lymphatic defects similar to those resulting from anti-ephrinB2 treatment (Fig. 3d). These findings demonstrated the functional importance of EphB4, and more importantly, they raised the possibility that EphB4-dependent forward signalling regulates lymphatic valve development. To explore this possibility, we examined the impact of antibody treatments on EphB4 activity *in vivo*. The activation status of EphB4 was monitored by its phosphorylation. In neonatal mice, EphB4 phosphorylation was significantly enhanced by anti-EphB4 (Supplementary Fig. 2b). In contrast, anti-ephrinB2 caused a rapid and dramatic reduction of EphB4 phosphorylation (Supplementary Fig. 2c). Furthermore, anti-EphB4 was able to restore the diminished EphB4 phosphorylation caused by anti-ephrinB2 (Supplementary Fig. 2d), which was in consistent with the ability of anti-EphB4 to rescue the lymphatic valve defect resulting from anti-ephrinB2. Together, these results provided further support to the functional significance of EphB4-dependent forward signalling.

To corroborate with the functional data, we investigated the expression of EphB4 by immunofluorescence staining. In P6 mouse mesenteries, strong EphB4 expression was detected in the veins while the expression in arteries and lymphatic vessels was relatively low. Interestingly, distinct expression of EphB4 was detectable in the lymphatic valve leaflets. Similarly, ephrinB2 expression was detected in lymphatic vessels and valves, although at lower level compared with that in arteries (Supplementary Fig. 5).

### Defective EphB4 forward signalling in *ephrinB2* mutants

**Figure 4:**
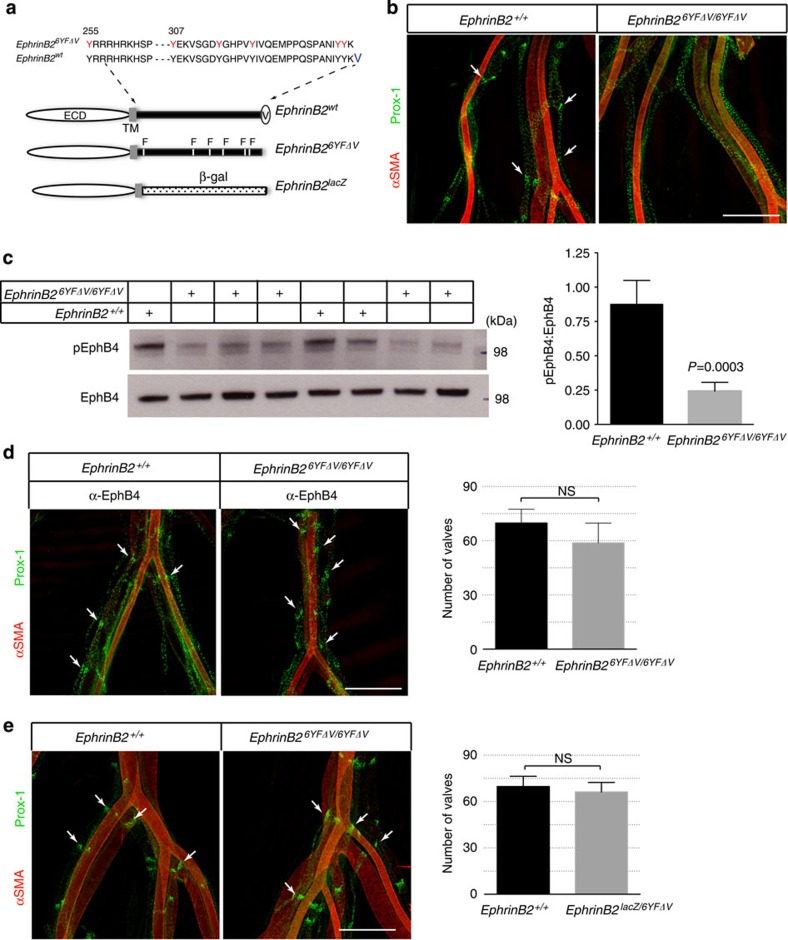
EphB4 activation rescues lymphatic valve defects in *ephrinB2* mutants. (**a**) Schematic representation of ephrinB2 mutants. (**b**,**d**,**e**) Visualization of mesenteric lymphatic vessels and valves (arrows) by immuno-staining for Prox-1. Strong α-smooth muscle actin (αSMA) staining highlights blood vessels. Scale bar, 500 μm. (**b**) Defective mesenteric lymphatic valve development in E18 *ephrinB2*^6YFΔV/6YFΔV^ embryos. (**c**) Compromised EphB4 activation in *ephrinB2*^6YFΔV/6YFΔV^ embryos. Total tissue lysates from E12.5 embryos were subjected to phospho-EphB4 (pEphB4) and total-EphB4 immunoblotting analysis. Ratios of pEphB4:EphB4 are graphed (right panel). (**d**) *In utero* treatment with agonist anti-EphB4 (α-EphB4) restores lymphatic valves in the mesentery of P0 *ephrinB2*^6YFΔV/6YFΔV^ mice. Right panel, quantification of mesenteric lymphatic valves, *n*=3 per genotype. (**e**) Normal mesenteric lymphatic valve development in *ephrinB2*^*lac*Z/6YFΔV^ neonatal mice (P3). Right panel, quantification of mesenteric lymphatic valves, *n*=3 per genotype. Statistical analysis in (**c**–**e**), two-tailed, unpaired Student's *t*-test, error bars, s.d. NS, not significant.

Our findings from the antibody treatment experiments also raised the possibility that the lymphatic defects caused by cytoplasmic mutations in ephrinB2 might be associated with impaired EphB4-forward signalling. We analysed a mouse line *ephrinB2*^6YFΔV/6YFΔV^, in which the six tyrosine residues in the cytoplasmic domain are changed to phenylalanine to eliminate tyrosine phosphorylation, and the C-terminal valine is deleted to prevent the interaction with PDZ domain-containing proteins^26^. Compared with *ephrinB2*^+/+^ embryos (E18), homozygous *ephrinB2*^6YFΔV/6YFΔV^ embryos failed to develop lymphatic valves in the leg skin (Supplementary Fig. 6a), similar to what has been reported in *ephrinB2*^ΔV/ΔV^ embryos^9^. Marked lymphatic valve defect was also observed in the mesenteric lymphatic vessels of *ephrinB2*^6YFΔV/6YFΔV^ embryos (Fig. 4b). We next examined the activation status of EphB4 in mouse embryos. EphB4 phosphorylation was significantly reduced in *ephrinB2*^6YFΔV/6YFΔV^ embryos as compared with *ephrinB2*^+/+^ embryos (Fig. 4c), indicating that *ephrinB2* mutant mice suffered compromised EphB4 forward signalling. We then examined the cell surface expression of ephrinB2 on lymphatic endothelial cells. EphrinB2 expression was comparable regardless of genotype. This result indicated that compromised EphB4 forward signalling was not simply due to reduced cell surface expression of ephrinB2 in *ephrinB2*^6YFΔV/6YFΔV^ embryos (Supplementary Fig. 7). The underlying molecular mechanism for this reduced *in vivo* EphB4 signalling induced by ephrinB2^6YFΔV^ remains to be investigated. Interestingly, it has been shown that the intracellular region of ephrinB can affect the signalling processing in EphB-expressing cells^27^.

### EphB4 activation rescues valve defects in *ephrinB2* mutants

If the lymphatic defects observed in *ephrinB2*^6YFΔV/6YFΔV^ embryos was due to impaired EphB4-dependent forward signalling, we reasoned that the agonistic EphB4 antibody might be able to reverse such defects. To perform the rescue experiment, we crossed the heterozygous *ephrinB2*^6YFΔV/+^ mice, and dosed the pregnant mice with anti-EphB4 starting at E12. Remarkably, we found that the lymphatic valves were prominently present in P0 mice of all genotypes born to the anti-EphB4-treated mice (Fig. 4d and Supplementary Fig. 6b). Together, our findings demonstrated that agonistic EphB4 antibody was able to rescue the lymphatic valve defects when ephrinB2 is either pharmacologically blocked or genetically compromised, further supporting the notion that EphB4-dependent forward signalling regulates lymphatic valve development.

### The intracellular region of EphrinB2 is dispensable

**Figure 5:**
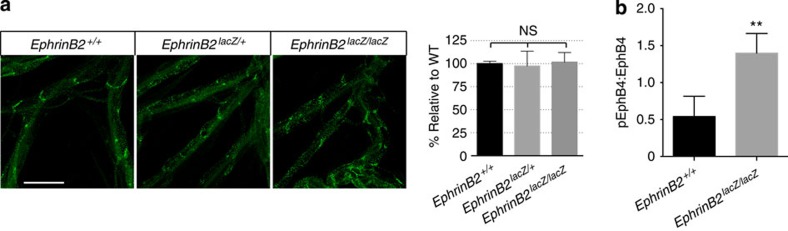
Lymphatic valves are present in *ephrinB2*^*lac*Z/*lacZ*^ mice. (**a**) Visualization of mesenteric lymphatic vessels and valves by immunostaining for Prox-1 in E18 embryos. Scale bar, 500 μm. Right panel, quantification of lymphatic valves, two-tailed, unpaired student's *t*-test, *n*=3 per genotype (error bars, s.d.). (**b**) Elevated EphB4 phosphorylaiton in *ephrinB2*^*lac*Z/*lacZ*^ embryos. Total tissue lysates from E12.5 embryos were subjected to phospho-EphB4 and total EphB4 immunoblotting analysis. Ratios of pEphB4:total EphB4 are graphed. **P<0.01 (two-tailed, unpaired student's *t*-test), *n*=6 for *ephrinB2*^+/*+*^, *n*=4 for *ephrinB2*^*lac*Z/*lacZ*^, error bars, s.d. NS, not significant.

The *ephrinB2*^*lac*Z^ allele, in which the entire cytoplasmic domain of ephrinB2 is replaced by β-gal, has also been used to interrogate the functional importance of ephrinB2-dependent reverse signalling^20^,^28^. As *ephrinB2*^*lac*Z/*lacZ*^ mice suffer perinatal lethality, we examined the lymphatic valves in E18 embryos. To our surprise, the lymphatic valves were abundantly present (Fig. 5a), which is in sharp contrast to *ephrinB2*^6YFΔV/6YFΔV^ mice that completely lack the lymphatic valves. In addition, *ephrinB2*^*lac*Z/*lacZ*^ mice do not phenocopy *ephrinB2*^6YFΔV/6YFΔV^ mice with respect to midline development^29^. As both *ephrinB2*^6YFΔV/6YFΔV^ and *ephrinB2*^*lac*Z/*lacZ*^ mice lack the critical signalling elements in the cytoplasmic region of ephrinB2, their distinct phenotypes argue against the view that compromised ephrinB2-dependent reverse signalling is the underlying cause of the observed developmental defects.

We crossed *ephrinB2*^*lac*Z/*+*^ and *ephrinB2*^6YFΔV/+^ mice to generate *ephrinB2*^*lac*Z/6YFΔV^ mutant mice. Although both *ephrinB2*^*lac*Z/*lacZ*^ and *ephrinB2*^6YFΔV/6YFΔV^ mice suffered perinatal lethality, *ephrinB2*^*lac*Z/6YFΔV^ mutant mice could live through adulthood. Moreover, lymphatic development in *ephrinB2*^*lac*Z/6YFΔV^ mice and wild-type littermates was comparable (Fig. 4e), indicating that the *ephrinB2*^*lac*Z^ allele and the *ephrinB2*^6YFΔV^ allele are compensating each other. Since ephrinB2^lacZ^ lacks the entire intracellular domain, and the all the key signalling elements are removed from ephrinB2^6YFΔV^, the apparently normal lymphatic development in *ephrinB2*^*lac*Z/6YFΔV^ mice suggested that the intracellular region of ephrinB2 is not required for lymphatic valve development.

In *ephrinB2*^*lac*Z/*lacZ*^ mice, it is likely that, due to the tetrameric nature of β-gal, ephrinB2-β-gal fusion protein is presented on cell surface in a highly clustered state, leading to enhanced EphB4 activation. Also it has been shown that the *ephrinB2*^*lac*Z^ allele exhibits elevated cell surface expression, and is genetically dominant in midline development^20^,^28^,^29^. Indeed, we found that EphB4 phosphorylation was significantly increased in *ephrinB2*^*lac*Z/*lac*Z^ embryos relative to *ephrinB2*^+/+^ embryos (Fig. 5b). This finding together with the observation of reduced EphB4 phosphorylation in *ephrinB2*^6YFΔV/6YFΔV^ embryos (Fig. 4c) suggests that *ephrinB2*^*lac*Z^ and *ephrinB2*^6YFΔV^ alleles are hypermorphic and hypomorphic, respectively, with respect to activating EphB4-dependent forward signalling during lymphatic valve development.

### Kinase activity of EphB4 is critical for valve development

**Figure 6:**
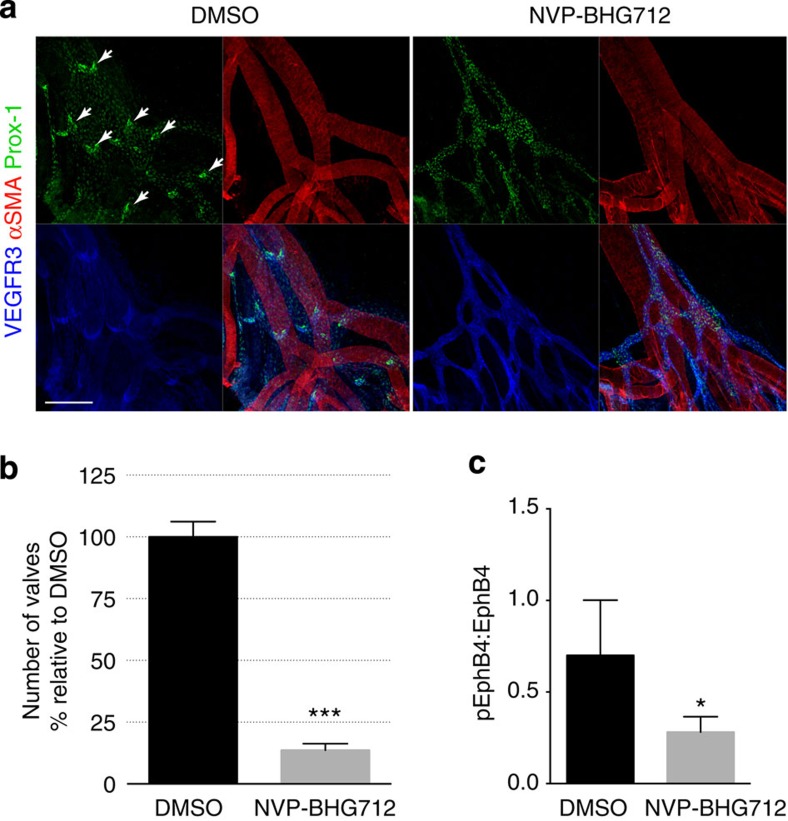
Kinase activity of EphB4 is required for lymphatic valve development. (**a**) Visualization of mesenteric lymphatic vessels and valves (arrows) by staining for Prox-1 and VEGFR3 2 days following treatment of P3 neonatal mice with NVP-BHG712, a selective EphB4 inhibitor. Blood vessels are highlighted by strong α-smooth muscle actin (αSMA) staining. Scale bar, 200 μm. (**b**) Quantification of mesenteric lymphatic valves, ***P< 0.001 (two-tailed, unpaired student's *t*-test), *n*=3 per treatment group (error bars, s.d.). (**c**) NVP-BHG712 inhibits EphB4 phosphorylation in P2 neonatal mice. Lung tissue lysates were subjected to anti-EphB4 immunoprecipitation followed by anti-pY or anti-EphB4 immunoblotting. Ratios of pEphB4 to total EphB4 (pEphB4: EphB4) are graphed. *P<0.05 (two-tailed, unpaired student's *t*-test), *n*=4 per treatment group (error bars, s.d.).

To assess the biological significance of EphB4-dependent forward signalling without any manipulation of ephrinB2, we utilized NVP-BHG712, a highly selective EphB4 kinase inhibitor^30^. We confirmed the potency of NVP-BHG712 inhibiting EphB4 phosphorylation in wild-type neonatal mice (Fig. 6c), and found that mice treated with NVP-BHG712 suffered marked loss of lymphatic valves (Fig. 6a,b), suggesting that the kinase activity of EphB4 is required for lymphatic valve formation and maintenance in early postnatal mice.

**Figure 7:**
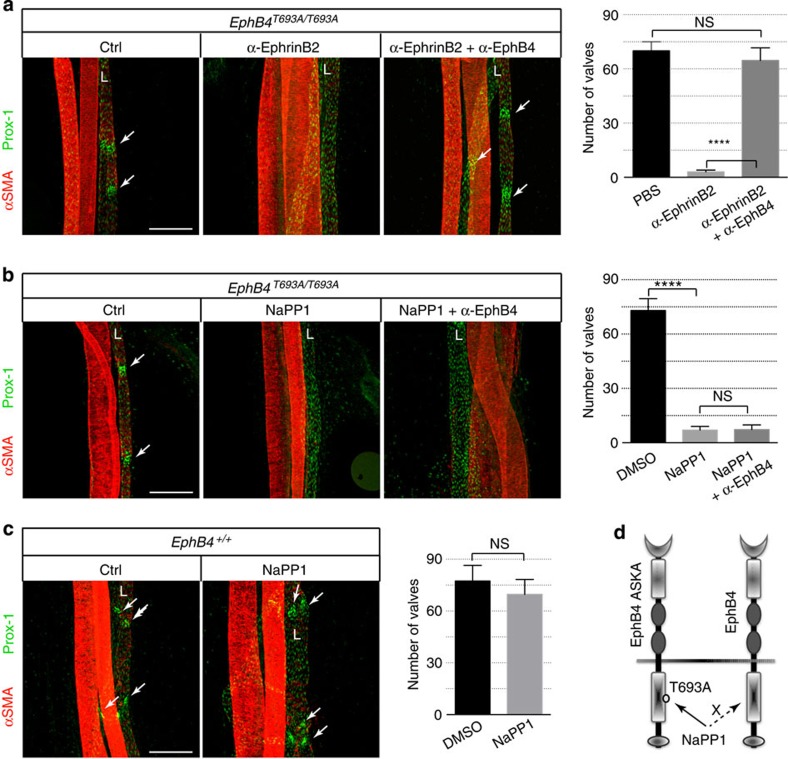
Chemical genetic inhibition of EphB4 in EphB4 ASKA mice. (**a**–**c**) Visualization of mesenteric lymphatic vessels (L) and valves (arrows) by immunostaining for Prox-1 following treatments starting from P2. Blood vessels are highlighted by strong α-smooth muscle actin (αSMA) staining. Scale bar, 200 μm. (**a**) Agonistic anti-EphB4 (α-EphB4) reverses lymphatic valve defects caused by function-blocking anti-ephrinB2 (α-ephrinB2) in EphB4 ASKA (*EphB4*^*T693A/T693A*^) neonatal mice. P7 mesenteric vessels are shown. Right panel, quantification of lymphatic valves. ****P<0.0001 (two-tailed, unpaired Student's *t*-test), *n*=3 per treatment group (error bars, s.d.). (**b**) NaPP1 treatment results in lymphatic valve defect in *EphB4*^*T693A/T693A*^ neonatal mice. P4 mesenteric vessels are shown. Right panel, quantification of lymphatic valves. ****P<0.0001(two-tailed, unpaired Student's *t*-test), *n*=3 per treatment group (error bars, s.d.). (**c**) NaPP1 treatment has no effect on lymphatic valves in wild-type neonatal mice. P4 mesenteric vessels are shown. Right panel, quantification of lymphatic valves, *n*=3 per treatment group, two-tailed, unpaired Student's *t*-test, error bars, s.d. (**d**) Schematic representation of EphB4 ASKA mutant (T693A). EphB4 ASKA is susceptible to inhibition by NaPP1. Ctrl, control; NS, not significant.

The analogue-sensitive kinase allele (ASKA) technology is a powerful tool for determining the *in vivo* function of individual kinases^31^,^32^. To exclude the possible off-target effect of NVP-BHG712, we took advantage of EphB4 ASKA (*EphB4*^*T693A/T693A*^) mice that are genetically engineered to carry a T693A mutation in the ATP-binding pocket, which sensitizes EphB4 to the inhibition by 1-Napthyl PP1 (NaPP1), an ATP-competitive inhibitor specifically designed to target mutant kinases. *EphB4*^*T693A/T693A*^ mice have no apparent developmental or postnatal defects, indicating that the T693A mutation does not affect the normal function of EphB4. Similar to wild-type mice, *EphB4*^*T693A/T693A*^ neonates displayed lymphatic valve defect after anti-ephrinB2 treatment, which was reversed when anti-EphB4 was co-administered (Fig. 7a). These results further confirmed that the T693A mutation did not affect the normal function of EphB4. Starting at P2, we treated the *EphB4*^*T693A/T693A*^ neonates with daily dosing of NaPP1. The treatment caused a dramatic loss of valve structures in mesenteric lymphatic vessels (Fig. 7b). In contrast, NaPP1 had little impact on wild-type neonates (Fig. 7c), indicating that the effect of NaPP1 in EphB4 ASKA mice was due to specific inhibition of EphB4 activity. In addition to the loss of lymphatic valves, EphB4 inhibition also caused lymphatic vessel dilation, similar to anti-ephrinB2 treatment (Supplementary Fig. 8). As Napp1 inhibits the kinase activity of a targeted kinase, our findings suggested that the kinase activity of EphB4 is critical for regulating lymphatic valve morphogenesis. Although anti-EphB4 was able to reverse the effect of anti-ephrinB2, it failed to rescue the lymphatic valve defect resulting from NaPP1 treatment (Fig. 7b), indicating that the agonistic activity of anti-EphB4 depends on the kinase activity of EphB4. Interestingly, NaPP1 treatment of EphB4 ASKA mice had no impact on EphrinB2 phosphorylation, suggesting that NaPP1 is specifically targeting EphB4 forward signalling in EphB4 ASKA mice (Supplementary Fig. 9). Taken together, these studies using two independent approaches to inhibiting EphB4 provide strong evidence that EphB4, in particular its kinase activity, is required for lymphatic valve formation and maintenance in neonatal mice.

## Discussion

Eph receptor tyrosine kinases and their plasma-membrane-anchored ephrin molecules control a broad spectrum of developmental processes by regulating cell migration and cytoskeletal organization in many cell types within different tissues. In the Eph-ephrin signalling system, in addition to the ‘forward' signalling mediated by Eph receptors, ephrins are capable of transmitting ‘reverse' signal via the tyrosine phosphorylation sites and a PDZ-binding motif in the cytoplasmic domain^12^,^16^.

Targeted inactivation of EphB4 and ephrinB2 has revealed the essential role EphB4–ephrinB2 interaction in regulating blood vascular development^14^,^24^,^25^. Unlike the *ephrinB2* null mice, mice carrying mutant ephrinB2 lacking the C-terminal PDZ interaction site (*ephrinB2*^ΔV/ΔV^) have normal blood vascular development, indicating that EphB4-dependent forward signalling but not ephirnB2-dependent reverse signalling regulates angiogenesis during early development. On the other hand, *ephrinB2*^ΔV/ΔV^ mice exhibit apparent defects in lymphatic remodelling and valve development, suggesting a role of ephrinB2 reverse signalling in lymphatic development^9^. The involvement of ephrinB2 signalling in lymphatic valve development was also reported in a recent paper by Katsuta H *et al*., showing that subconjunctival injection of EphB4-Fc resulted in the deformation of preexisting corneal lymphatic valves in the mouse eye^33^. EphB4-Fc used in their study worked as a ligand trap to prevent ephrinB2 from interacting with EphB4 or other Eph receptors.

Our current study specifically investigated the role of EphB4 forwarding in lymphatic valve development. We developed highly selective EphB4 agonist (bivalent anti-EphB4) or antagonist (monovalent anti-EphB4 Fab). Neonatal mice treated with antagonistic anti-EphB4 Fab developed lymphatic valve defects similar to those observed in *ephrinB2*^ΔV/ΔV^ mice or WT mice treated with a functional blocking anti-ephrinB2 antibody. In contrast, direct activation of EphB4 using the agonistic anti-EphB4 antibody rescued the lymphatic valve defect when ephrinB2 was either pharmacologically blocked or genetically compromised. Besides the antibody tools that target receptor–ligand interaction, we used small-molecule inhibitors that target the kinase activity of EphB4. We found that NVP-BHG712, a highly selective EphB4 kinase inhibitor caused defective lymphatic valve development in neonatal mice. To exclude the possible off-target effect of NVP-BHG712, we further employed a completely different approach to inhibit the kinase activity of EphB4. We took advantage of a chemical genetic approach using EphB4 ASKA (*EphB4*^*T693A/T693A*^) mice, which allowed the functional assessment of a specific protein kinase *in vivo*. The results of these studies clearly defined a role of EphB4 forward signalling in lymphatic valve development.

The biological significance of ephrinB reverse signalling has largely been derived from genetic experiments employing mice carrying mutations in the intracellular regions of ephrinBs. In the current study, we tested two different mouse lines that express mutant ephrinB2 with an altered intracellular region. The *ephrinB2*^6YFΔV/6YFΔV^ mice express a mutant ephrinB2 in which the six tyrosine residues in the cytoplasmic domain are changed to phenylalanine to eliminate tyrosine phosphorylation, and the C-terminal valine is deleted to prevent the interaction with PDZ domain-containing proteins. Similar to *ephrinB2*^ΔV/ΔV^mice^9^, *ephrinB2*^6YFΔV/6YFΔV^ mice exhibited marked lymphatic valve defect. Interestingly, we found that EphB4 phosphorylation was significantly reduced in *ephrinB2*^6YFΔV/6YFΔV^ embryos as compared with *ephrinB2*^+/+^ embryos. Because the cell surface expression of ephrinB2 was comparable between WT and mutant ephrinB2, this result suggested that ephrinB2^6YFΔV/6YFΔV^ has a compromised signalling capacity in activating EphB4. This finding was reminiscent of a report showing that the intracellular region of ephrinB can affect the signalling processing in EphB-expressing cells^27^, although the underlying molecular mechanism remains unclear. The *ephrinB2*^*lac*Z/*lacZ*^ mice express a mutant ephrinB2 in which the entire cytoplasmic domain of ephrinB2 is replaced by β-gal. The mutant mice suffer various developmental abnormalities, including problems with cardiac valve development and urogenital-anorectal development, defective cleft palate and failed tracheoesophageal separation^20^,^28^,^29^. All these defects could contribute to the perinatal lethality. We found that EphB4 phosphorylation was significantly increased in *ephrinB2*^*lac*Z/*lac*Z^ embryos relative to *ephrinB2*^+/+^ embryos, suggesting that ephrinB2–β-gal fusion protein has increased capacity in activating EphB4. Therefore, the developmental defects observed in the *ephrinB2*^*lac*Z/*lacZ*^ mice could be the consequence of defective ephrinB2 reverse signalling that depends on its intracellular domain, or the result of abnormally elevated forward signalling through Eph receptors. Interestingly, unlike the *ephrinB2*^6YFΔV/6YFΔV^ mice, which completely lack lymphatic valves, lymphatic valves were abundantly present in the *ephrinB2*^*lac*Z/*lacZ*^ mice. Furthermore, we found completely normal lymphatic development in the *ephrinB2*^*lac*Z/6YFΔV^ mice, in which the *ephrinB2*^*lac*Z^ allele and the *ephrinB2*^6YFΔV^ allele are likely compensating each other. These findings suggest, but do not prove, that the intracellular region of ephrinB2 is not required for lymphatic valve development. It should be pointed out that the *ephrinB2*^*lac*Z/6YFΔV^ mice still have other developmental defects^29^, indicating a role of ephrinB2 reverse signalling in those developmental processes.

In summary, our work definitely established a role of EphB4-dependent forward signalling in lymphatic valve development. In addition, our findings open a new perspective for developing potential therapeutics using function-activating or -blocking antibodies that selectively target individual Ephs and ephrins.

## Methods

### Generation of phage antibodies

Anti-ephrinB2 and anti-EphB4 were isolated from synthetic phage antibody libraries built on a single framework by introducing diversity within the complementarity-determining regions (CDRs) of heavy and light chains^34^,^35^. Plate panning with naïve libraries was performed against His-tagged human ephrinB2 (amino acids 1-174) or human EphB4 (amino acids 1–539). The resulting clones were further screened with His-tagged murine ephrinB2 (amino acids 1–174) or murine EphB4 (amino acids 1–539) to identify cross-species clones. For affinity maturation, phage libraries with three different combinations of CDR loops (CDR-L3, -H1 and -H2) derived from the initial clone of interest were constructed by soft randomization strategy. High-affinity clones were then identified through several rounds of solution phase panning with progressively increased stringency. Variable regions of heavy and light chains from the finally identified phage clones were subcloned into mammalian expression vectors engineered to express full-length IgG chains. Heavy chain and light chain constructs were co-transfected into 293 or CHO cells, and the expressed antibodies were purified from serum-free medium using protein-A affinity column. Binding kinetics of purified antibody was measured by BIAcore-3000 instrument. Association rates (*k*_on_) and dissociation rates (*k*_off_) were calculated using a simple one-to-one Langmuir-binding model (BIAcore Evaluation Software version 3.2). The equilibrium dissociation constant (Kd) was calculated as the ratio *k*_off_/*k*_on_.

### Cell cultures

HUVECs were obtained from Cambrex Clonetics Cell Systems. 3T3-ephrinB2 and 3T3-EphB4 cell lines were established by selecting stable clones from 3T3 cells transfected with plasmids expressing human ephrinB2 and human EphB4, respectively.

### Western blot analysis of EphB4 phosphorylation

EphB4 phosphorylation in HUVEC cells was induced by overlaying 3T3 cells for 15 min. EphB4 phosphorylation in EphB4-expresing 3T3 cells was stimulated by anti-EphB4 (Genentech, 5 μg ml^−1^) or ephrinB2-Fc (5 μg ml^−1^) for 10 min. The cells were lysed in NP-40 lysis buffer (50 mM Tris (pH 7.5), 150 mM NaCl, 1% NP-40) supplemented with Protease Inhibitor Cocktail (Sigma, Cat. # p8340) and Phosphatase Inhibitor Cocktail 2 (Sigma, Cat. # 5726). Cell lysates were clarified and subjected to immunoprecipitation using phage-derived anti-EphB4 antibody (Genentech, 5 μg ml^−1^), followed by immunoblotting with anti-phosphotyrosine antibody (Sigma, clone 4G10, 1 μg ml^−1^), or anti-EphB4 (Santa Cruz, Cat. # sc-5536, 1 μg ml^−1^). The bound antibodies were detected using horseradish peroxidase (HRP)-conjugated secondary antibodies (Jackson ImmunoResearch, 1:20,000) and Enhanced chemiluminescence (ECL). The full western blot data are shown in supplementary Fig. 10.

### ELISA

To determine the selectivity of anti-EphB4, 96-well microtitre plates were coated with the Fc fusion recombinant proteins of the extra cellular domains of EphB1, EphB2, EphB3, EphB4 and EphB6 (R&D system, 5 μg ml^−1^) in PBS. Plates were then washed and blocked with PBS and 0.5% BSA. Biotinylated anti-EphB4 (Genentech, 0.1 μg ml^−1^) was added. Plates were washed 3 × with binding buffer and then probed with streptavidin-HRP (Jackson ImmunoResearch, 1 μg ml^−1^). HRP activity was developed using Sure Blue Reserve TMP Microwell Peroxidase Substrate (Kirkegaard & Perry Laboratories, Inc), and absorbance was measured at 450 nm. An identical procedure was used to determine the selectivity of anti-ephrinB2 except that the recombinant proteins of the extra cellular domains of ephrinB1, ephrinB2 and ephrinB3 (R&D system, 5 μg ml^−1^), and biotinylated anti-ephrinB2 (Genentech, 0.1 μg ml^−1^) were used. We also generated ephrinB2-AP and EphB4-AP, which contain the extracellular domain of ephrinB2 and EphB4, respectively, fused to human placenta alkaline phosphatase. Cell culture medium from 293 cells transfected with plasmid-expressing ephrinB2-AP or EphB4-AP (1:4 dilution) was used for assessing the blocking activities of anti-ephrinB2 and anti-EphB4.

### Visualization of lymphatic vasculature

FITC-dextran (Molecular probe, Cat. # D7137, 10 mg ml^−1^ in PBS) or FITC-lectin (Vector Laboratories, Inc., Cat. # FL-1171, 1 mg ml^−1^ in PBS) was injected subcutaneously (5 μl each foot pad) into an anaesthetized mouse to allow circulation for 10–30 min, and lymphatic vessels and valves were examined by fluorescence or confocal microscopy (Zeiss LSM 510).

### Immunostaining of lymphatic and blood vessels

For whole-mount staining, the tissues were fixed in 4% paraformaldehyde 2–4 h, and blocked in 2% Chicken serum, 1% BSA, 0.1% cold fish skin gelatin, 0.5% Tx100, 0.05% Tween-20, 0.05% Azide in PBS. The primary antibodies are rabbit antibody to mouse Prox-1 (Fitzgerald, Cat. # 70R-PR039, 1 μg ml^−1^), Cy3-conjugated mouse antibody to mouse α-smooth muscle actin Sigma, Cat. # C6189, 1:400), and goat antibody to mouse VEGFR3 (R&D Systems, Cat. # AF743, 1 μg ml^−1^). Secondary antibodies are Alexa Fluor 488 Chicken anti-rabbit (Invitrogen, Cat. # A21441, 1:500) and Alexa Fluor 647 Chicken anti-Goat (Invitrogen, Cat. # A21469, 4 μg ml^−1^). The stained tissues were analysed using Zeiss LSM 510 laser scanning confocal microscope.

### Animal studies and mouse lines

All studies were conducted in accordance with the National Institutes of Health Guide for the Care and Use of Laboratory Animals (NIH Publication 85–23, revised 1985). An Institutional Animal Care and Use Committee approved all animal protocols. *ephrinB2*^*lac*Z/*lacZ*^ and *ephrinB2*^6YFΔV/6YFΔV^ mice have been described previously^26^,^28^. EphB4 ASKA (*EphB4*^*T693A/T693A*^) mice were obtained from Taconic. All neonatal experiments use mixed sex mice. The age of mice used in each study is specified in figure legends.

### Western blot analysis of *in vivo* EphB4 phosphorylation

Mouse lung or whole embryo lysates were prepared using CHAPS lysis buffer (FIVEphoton Biochemicals) supplemented with phosphatase inhibitor cocktails (Sigma, 1:100) and proteinase inhibitors (Roche). EphB4 was immunoprecipitated with phage anti-EphB4 antibody (Genentech, 5 μg ml^−1^), followed by immunoblotting with anti-phosphotyrosine antibody (Sigma, clone 4G10, 1:1,000) or anti-EphB4 (R&D Systems, 1 μg ml^−1^). The full western blot data are shown in supplementary Fig. 10.

### Western blot analysis of *in vivo* EphrinB2 phosphorylation

Mouse lung lysates were prepared using CHAPS lysis buffer (FIVEphoton Biochemicals) supplemented with phosphatase inhibitor cocktails (Sigma, 1:100) and proteinase inhibitors (Roche). EphrinB2 was immunoprecipitated with phage anti-EphrinB2 antibody (Genentech, 5 μg ml^−1^), followed by immunoblotting with anti-phospho (Y316) EphrinB2 antibody (Abcam, ab119323, 1 μg ml^−1^) or Biotin-anti-EphrinB2 (R&D Systems, Cat#BAF496, 0.4 μg ml^−1^).

### Small-molecule inhibitor of EphB4

NVP-BHG712, a small-molecule weight kinase inhibitor of EphB4, was purchased from Santa Cruz Biotechnology (Cat # sc-364554). To examine the effect of inhibition of EphB4 kinase activity on lymphatic vessels, P3 neonatal mice were administered with a daily IP dose of NVP-BHG712 at 50 mg kg^−1^ or vehicle control (DMSO). Lymphatic vessels were examined at P5. To examine the effect of EphB4 inhibition in EphB4 ASKA mice, P2 neonatal mice were treated with a daily IP dose of NaPP1 (10 mg kg^−1^) or vehicle control (DMSO). Lymphatic vessels were examined at P4.

### Quantification and statistics

The number of lymphatic valves was quantified using five mesenteric collecting vessels per mesentery in three or four mice of each genotype or treatment group. Groups were compared using a two-tailed, unpaired Student's *t*-test. *P* values <0.05 were considered significant. Data are shown as mean±s.d.

## Author contributions

G.Z. and M.Y. designed the experiments. G.Z. performed the animal, biochemical and histological experiments. J.R. performed the biochemical and molecular experiments. Y.W. and W.-C.L. the generated antibodies. M.H. provided the mouse lines and technical advice. G.Z. and M.Y. wrote the manuscript.

## Additional information

**How to cite this article:** Zhang G. *et al*. EphB4 forward signalling regulates lymphatic valve development. *Nat. Commun.* 6:6625 doi: 10.1038/ncomms7625 (2015).

## Supplementary Material

Supplementary Information Supplementary Figures 1-10
