# Supplementary material for: EphB4 forward signalling regulates lymphatic valve development
Source: Nat Commun. 2015 Apr 13;6:6625. doi: 10.1038/ncomms7625 (PMC4403310; doi:10.1038/ncomms7625)
Supplement: Supplementary Information — Supplementary Figures 1-10 [file ncomms7625-s1.pdf]

Supplemental Information

EphB4 forward signaling regulates lymphatic valve development

Gu Zhang[1], John Brady[1], Wei-Ching Liang[2], Yan Wu[2], Mark Henkemeyer[3], and Minhong Yan[1,4]

[1]Department of Molecular Oncology, [2]Department of Antibody Engineering, Division of Research, Genentech Inc., 1 DNA Way, South San Francisco, CA 94080, USA, [3]Department of Developmental Biology, University of Texas Southwestern Medical Center, Dallas, TX 75390, USA

[4]Corresponding author. Email minhong@gene.com

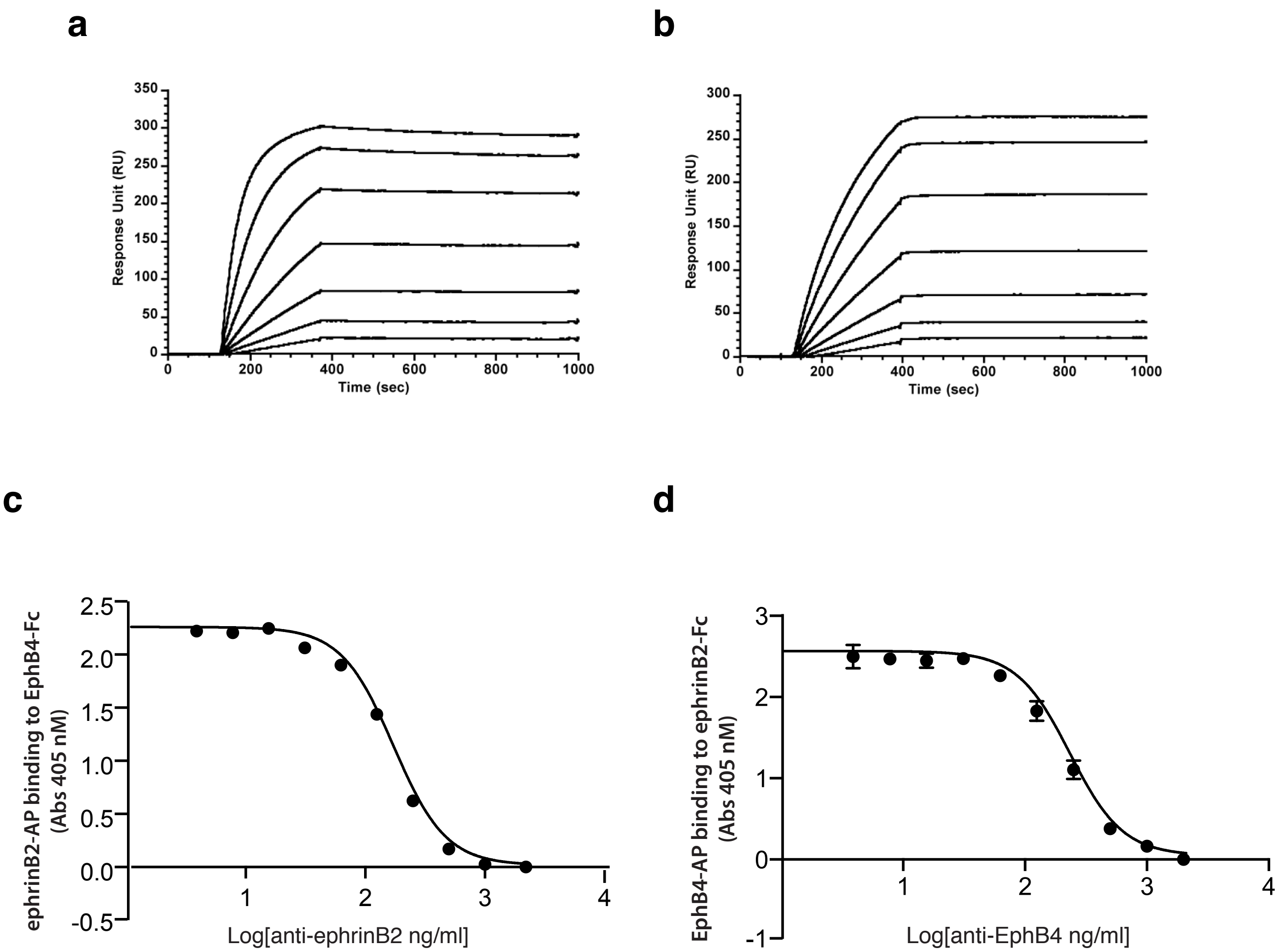

Supplementary Figure 1 | Characterization of anti-ephrinB2 and anti-EphB4 antibodies. (**a**, **b**) Binding kinetics of anti-ephrinB2 and anti-EphB4 measured by Plasmon Resonance (SRP). (**a**) Anti-ephrinB2: Kon =7 x 10^5 (1/Ms), Koff = 5.8 x 10^{-5} (1/s), Kd = 83 pM. (**b**), Anti-EphB4: Kon= 4 x 10^4 (1/Ms), Koff $\leq$ 5 x 10^{-6} (1/s), Kd $\leq$ 125 pM. (**c**) Dose-dependent blocking of ephrinB2-AP binding to EphB4-Fc by anti-ephrinB2. (**d**) Dose-dependent blocking of EphB4-AP binding to ephrinB2-Fc by anti-EphB4. Error bars, SD of technical duplicates.

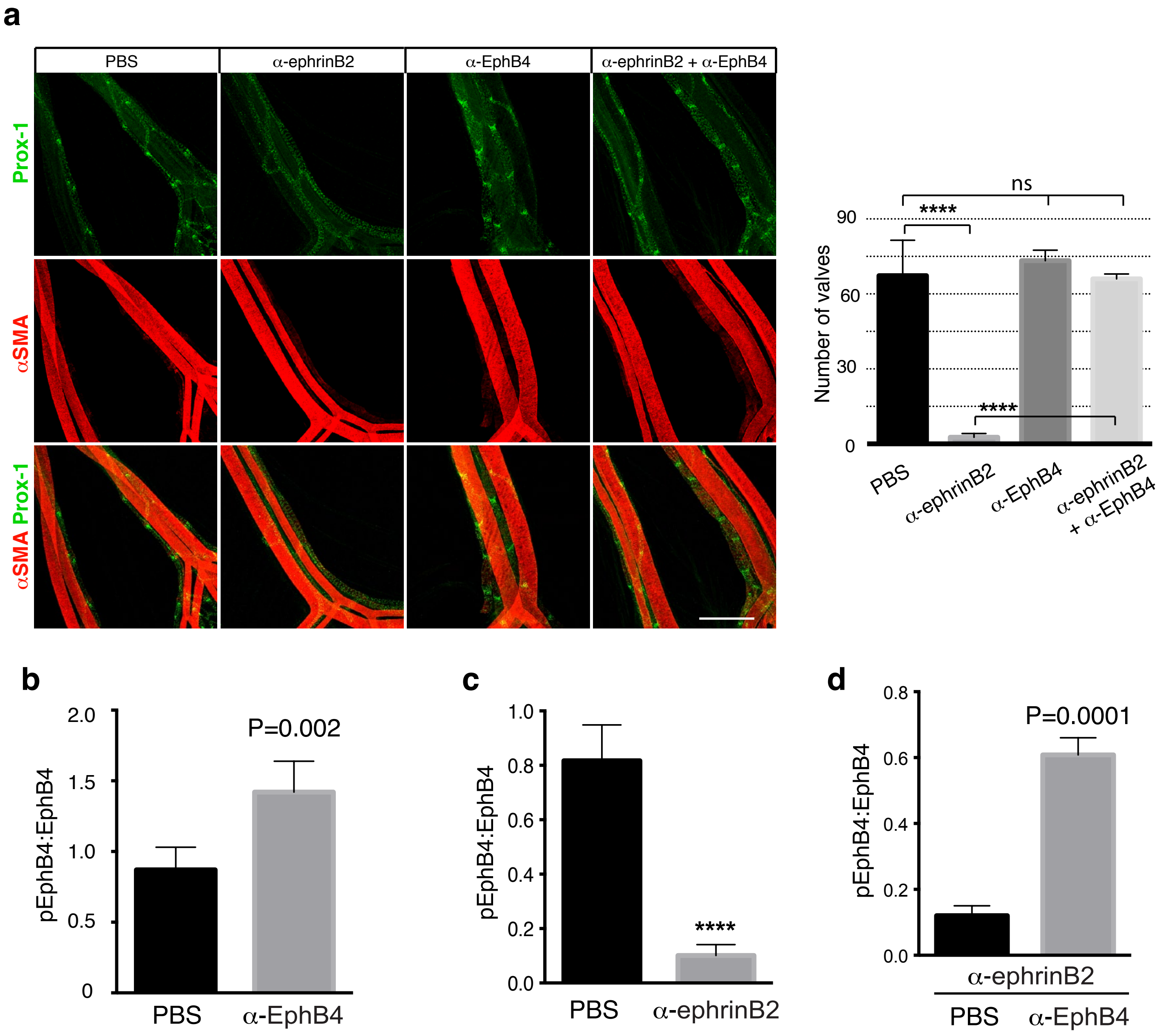

Supplementary Figure 2 | (**a**) **Anti-EphB4 rescues the lymphatic valve defects caused by anti-ephrinB2.** P6 mesenteric lymphatic vessels and valves are visualized by immunostaining for Prox-1 following antibody treatment starting from P1, at 10 mg/kg, every two days. Blood vessels are high-lighted by strong αSMA staining. Scale bar, 500 μm. Right panel, quantification of mesenteric lymphatic valves. (****)p< 0.0001(two-tailed, unpaired student's *t*-test), n=3 per treatment group (error bars, SD).(**b-d**) **Effect of antibody treatment on EphB4 phosphorylation in neonatal mice (P2)**. Lung tissue lysates were subjected to anti-EphB4 immunoprecipitation followed by anti-pY or anti-EphB4 immunoblotting. Ratios of pEphB4 (phospho-EphB4) to total EphB4 (pEphB4: EphB4) are graphed. (**b**) Anti-EphB4 elevates EphB4 phosphorylation. Lung tissues were collected 45 min after antibody injection. Two-tailed, unpaired student's *t*-test, n=5 per treatment group (error bars, SD). (**c**) Anti-ephrinB2 reduces EphB4 phosphorylation. Lung tissues were collected 120 min after antibody injection. (****)P< 0.0001 (two-tailed, unpaired student's *t*-test), n=4 per treatment group (error bars, SD). (**d**) Anti-EphB4 restores EphB4 phosphorylation reduced by anti-ephrinB2. All mice were treated with anti-ephrinB2 for 120 min, followed by either control (PBS) or anti-EphB4 injection for 45 min. Two-tailed, unpaired student's *t*-test, n=3 per treatment group (error bars, SD).

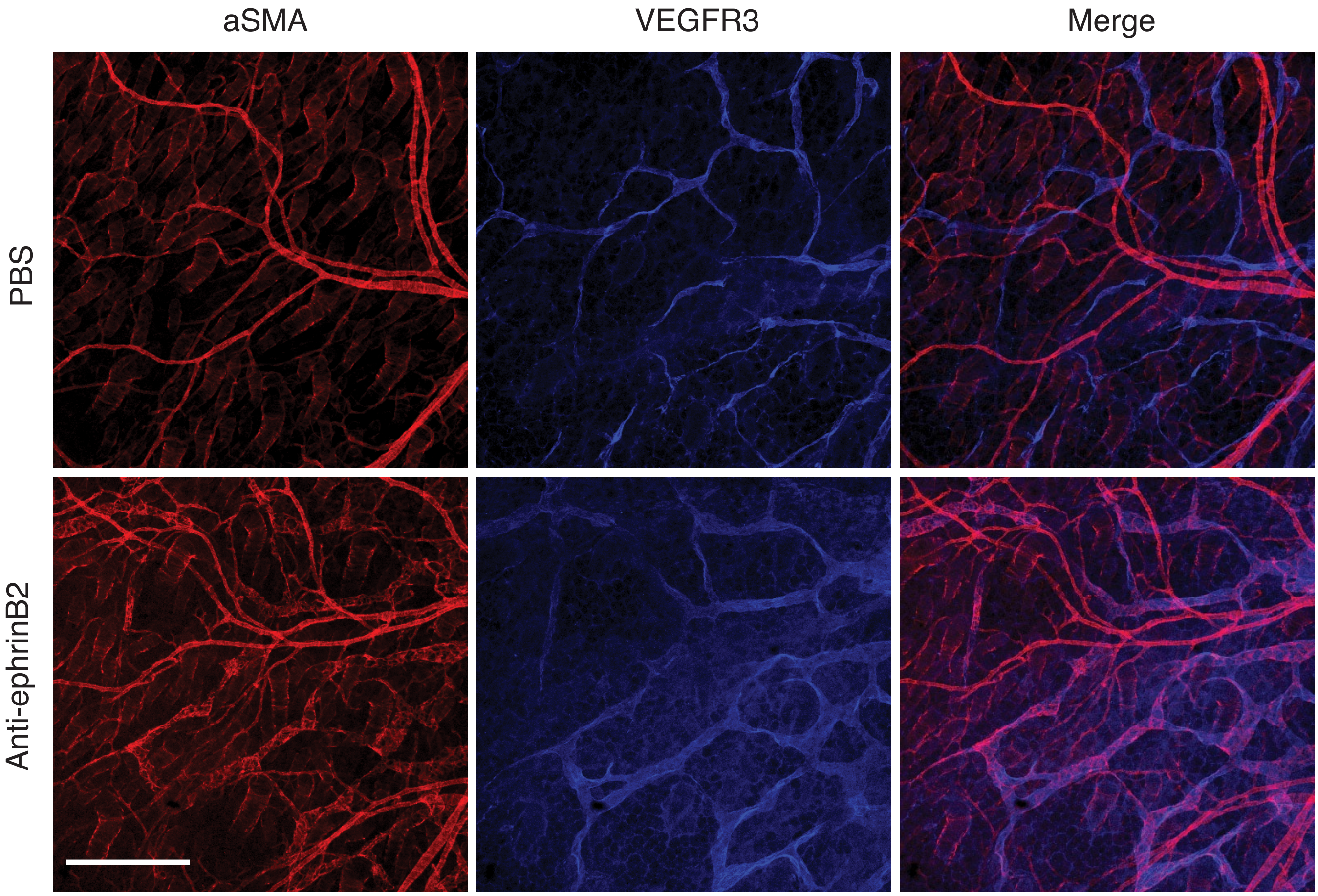

Supplementary Figure 3 | Treatment of anti-EphrinB2 antibody resulted in significant increase of smooth muscle cell coverage in lymphatic capillaries. Neonatal mice were treated with anti-EphrinB2 antibody at P1, P3 and P5. P6 leg skins were stained with aSMA and VEGFR3. Scale bar, 500 µm.

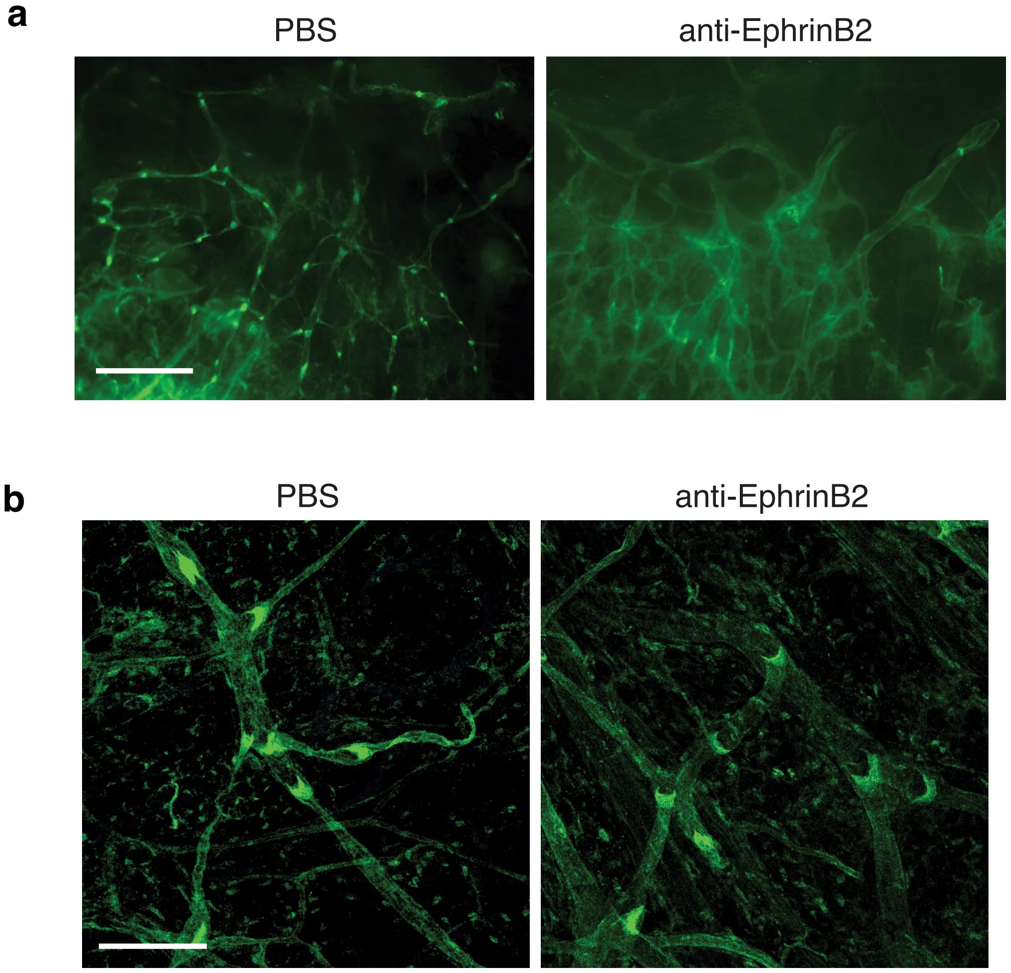

Supplementary Figure 4 | EphrinB2 signaling is not required for lymphatic valve maintenance in adult mice. (**a, b**) Visualization of ear skin lymphatic vessels and valves by FITC-lectin. (**a**) Ear skin of P7 mice. Neonatal CD1 mice were treated with anti-EphrinB2 at P1, P3 and P5. Scale bar, 500 µm. (**b**) Ear skin of 10-week-old female mice. 8-week-old female CD1 mice were treated with anti-EphrinB2, twice per week for two weeks. Scale bar, 200 µm.

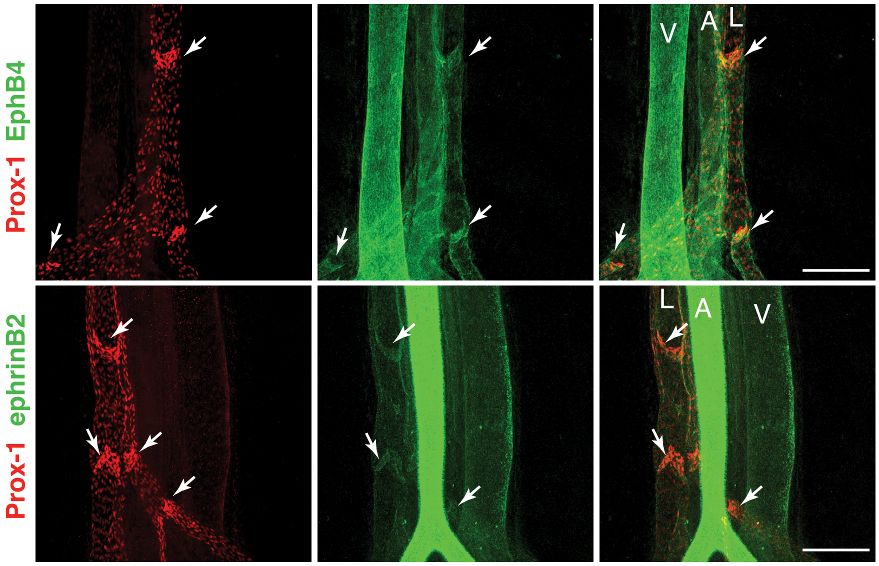

Supplementary Figure 5 | Expression of EphB4 and ephrinB2 in lymphatic vessels. Mesenteric lymphatic vessels of P6 mice are immunostained for Prox-1, together with either EphB4 (upper panel) or ephrinB2 (lower panel). Lymphatic valves are marked with arrows. V, vein; A, artery; L, lymphatic. Scale bar, 200 µm.

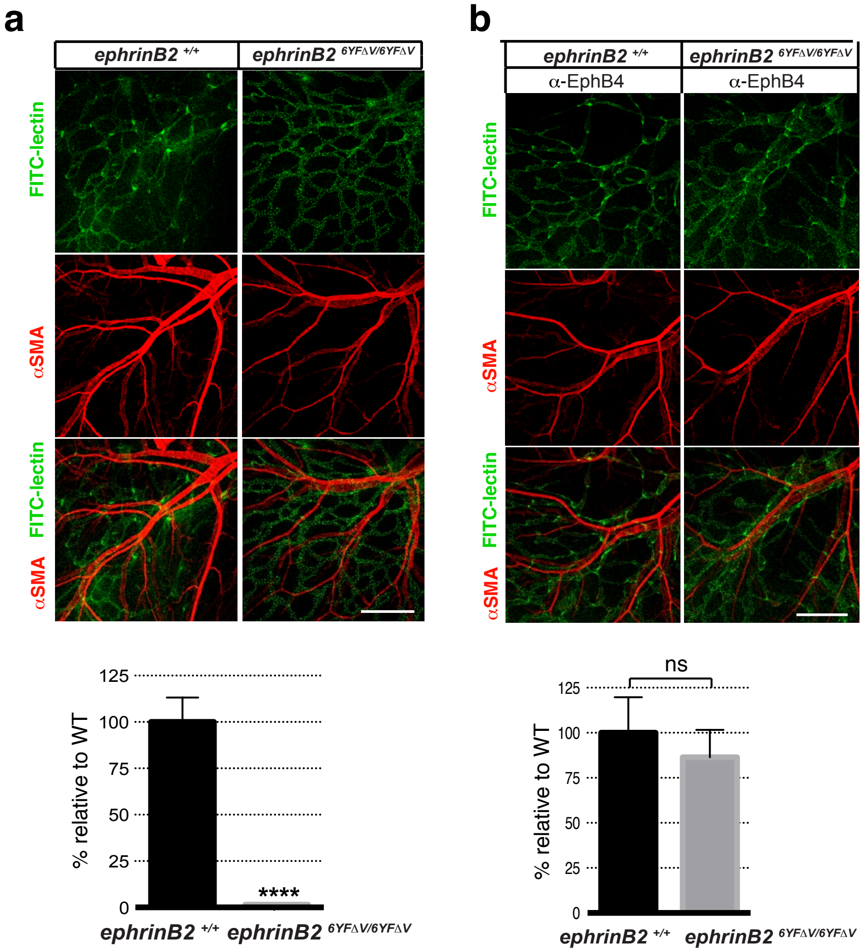

Supplementary Figure 6 | (**a**,**b**) Visualization of leg skin lymphatic vessels and valves by FITC-lectin. Strong αSMA staining highlights blood vessels. Scale bar, 500 µm. (**a**) Defective lymphatic valve development in the leg skin of E18 *ephrinB2* $^{6YF\,\Delta V/6YF\Delta V}$ embryos. Lower panel, quantification of lymphatic valves, (****)p< 0.0001(two-tailed, unpaired student's *t*-test), n=4 per genotype (error bars, SD). (**b**) *In utero* treatment with agonist α-EphB4 restores leg skin lymphatic valves in P0 *ephrinB2*$^{6YF\,\Delta V/6YF\,\Delta V}$ mice. Lower panel, quantification of lymphatic valves, two-tailed, unpaired student's *t*-test, n=3 per genotype (error bars, SD).

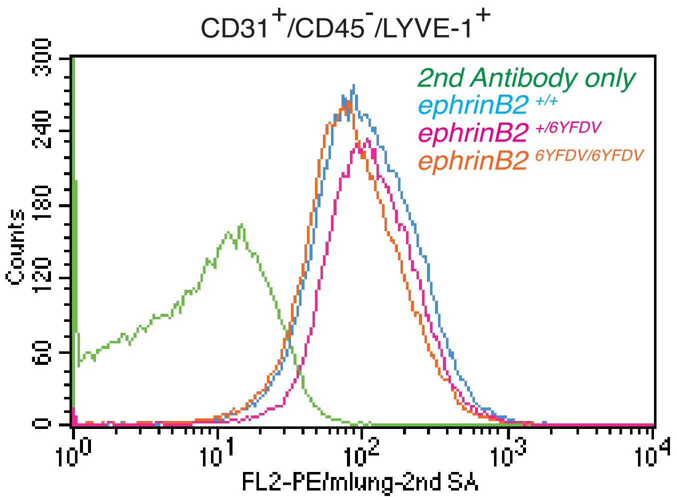

Supplementary Figure 7 | FACS analysis of cell surface expression of EphinB2 on lung lymphatic endothelial cells. Single cell suspensions were prepared from E18 lung tissues. Analysis of EphrinB2 expression was gated on $CD31^+/CD45^-/LYVE\text{-}1^+$ lymphatic endothelial cells.

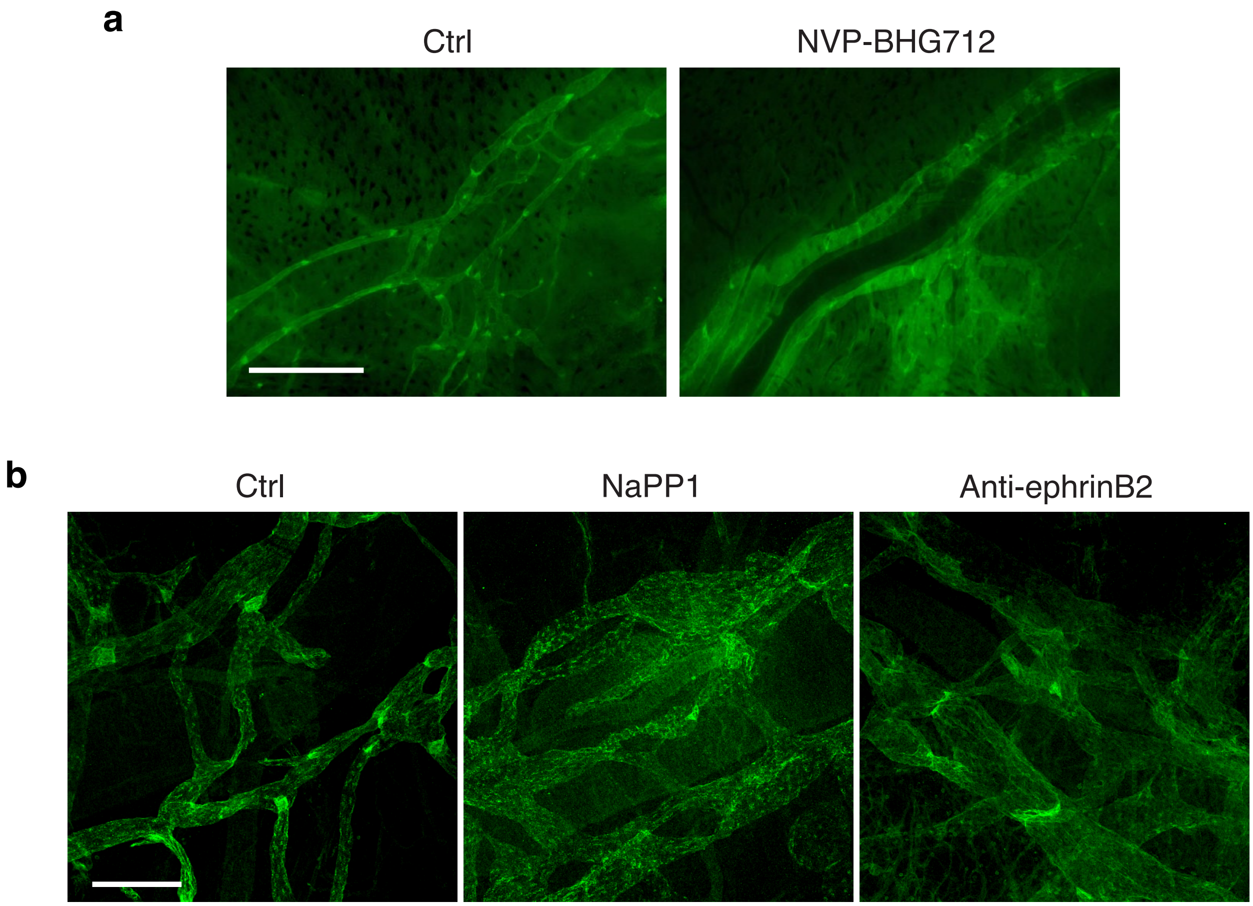

Supplementary Figure 8 | Inhibition of EphB4 kinase activity results in dilated lymphatic vessels. Lymphatic vessels and valves were visualized by footpad injection of FITC-lection. (**a**) Neonatal mice were treated with NVP-BHG712 daily starting from P2, and leg lymphatic vessels were examined at P5. Scale bar, 500 µm. (**b**) Neonatal EphB4 ASKA mice were treated with NaPP1 or anti-ephrinB2 daily starting from P2, and leg lymphatic vessels were examined at P5. Scale bar, 200 µm.

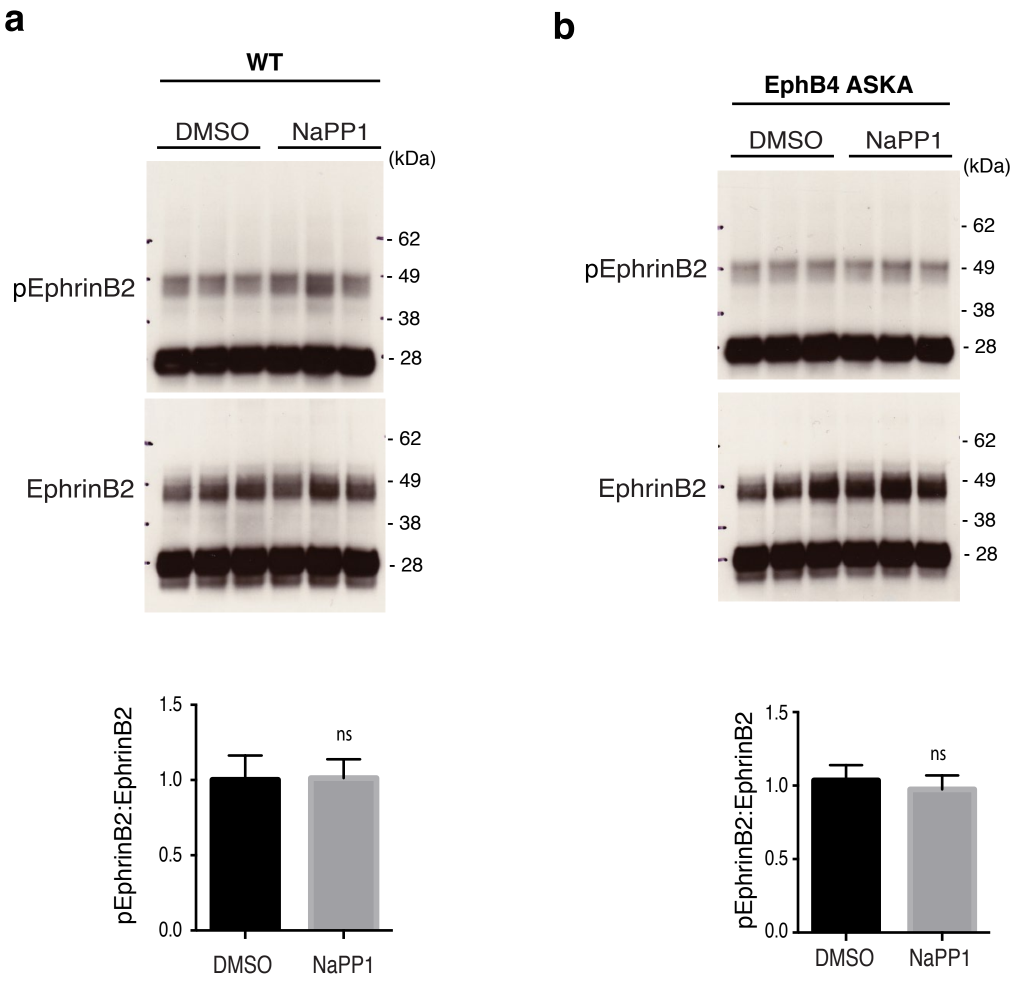

Supplementary Figure 9| EphB4 inhibition in EphB4 ASKA mice does not affect EphrinB2 phosphorylation. WT mice (**a**) or EphB4 ASKA mice (**b**) were daily treated with DMSO or NaPP1 starting from P2. P5 lung tissues were subjected to phospho-EphrinB2 (pEphrinB2) and total-EphrinB2 immunoblotting analysis. Ratios of pEphrinB2:EphrinB2 are graphed. Two-tailed, unpaired student's *t*-test, n= 3 per treatment group (error bars, SD).

Fig.1b

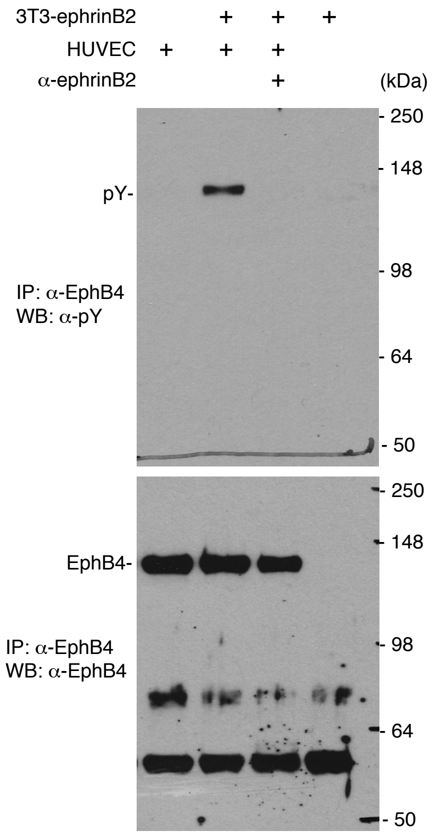

Fig.1c

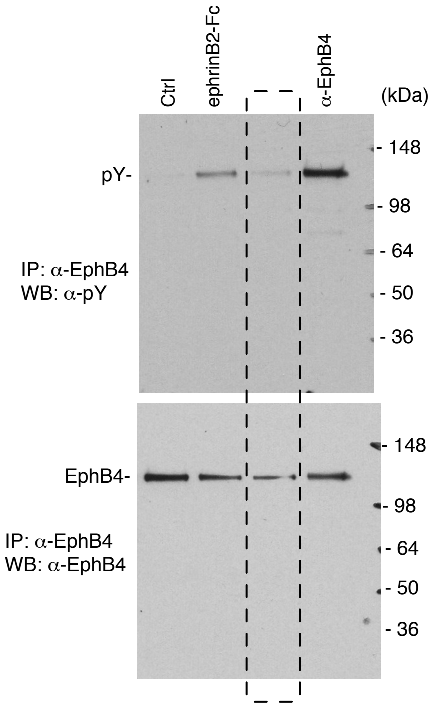

Fig. 4c

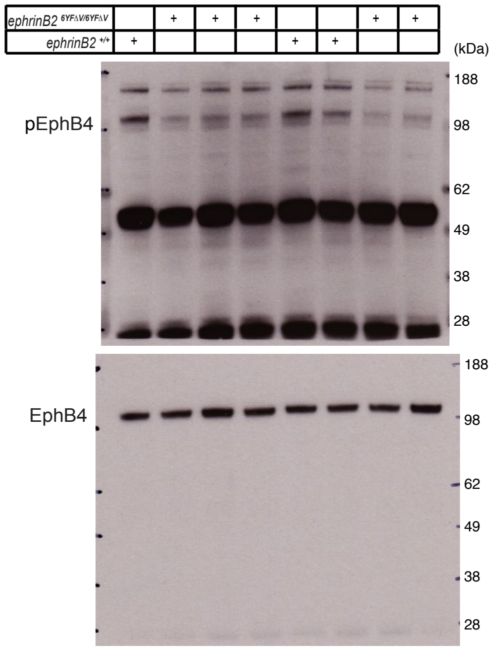

Supplementary Figure 10 | Full Western blot data
